# Burden of treatment in vulval lichen sclerosus

**DOI:** 10.1002/ski2.125

**Published:** 2022-05-16

**Authors:** Ciara O’Grady, Cathal O’Connor, Aysha Al Moosa, Michelle Murphy, Eilis Nic Dhonncha

**Affiliations:** ^1^ Dermatology South Infirmary Victoria University Hospital Cork Ireland; ^2^ Medicine University College Cork College Road Cork Ireland

## Abstract

Lichen sclerosus (LS) is a chronic inflammatory dermatosis predominantly affecting the anogenital region, which can have significant impact on quality of life. Burden of treatment (BOT) is defined as the workload of healthcare experienced by patients and consequences on well‐being. In this prospective study, 35 women with vulval LS completed a detailed Treatment Burden Questionnaire to assess their BOT. Nineteen (54.3%) achieved a score of 35 or less, signifying low BOT; ten (28.6%) between 36 and 65, signifying moderate BOT; and six (17.1%) above 65, signifying high BOT. Seven (20%) patients reported BOT scores of greater than 59, which has been designated as a cut‐off for increased risk of treatment‐related burnout. Higher BOT scores were moderately correlated with higher DLQI scores (r = 0.47, p < 0.01). BOT in LS is low for most patients, although a minority are at risk of treatment‐related burnout. BOT should be considered when forming treatment guidelines for LS.

## Graphical abstract

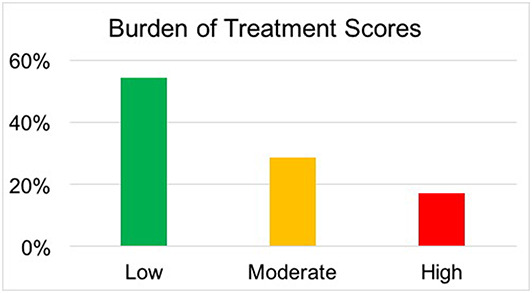

1

Dear Editor,

Lichen sclerosus (LS) is a chronic inflammatory condition predominantly affecting the anogenital region, which can have significant impact on patients' quality of life.^1^ For vulval LS, prolonged treatment with ultrapotent topical corticosteroids (TCS), for example, clobetasol propionate 0.05%, is a mainstay of therapy; to maintain disease control, to prevent progression, and to potentially reduce the risk of developing an associated squamous cell carcinoma.^2^ Patients with vulval LS may be managed by dermatologists, gynaecologists, urologists, primary care physicians, or paediatricians. In Ireland, vulval LS is typically managed by dermatologists. Burden of treatment (BOT) is defined as the workload of healthcare experienced by those with chronic conditions and consequences on well‐being.^3^ While it has been shown that patients with LS have moderate satisfaction with therapy,^1^ no studies have investigated the burden of treatment in LS. The aim of this study was to assess the BOT in women with vulval LS.

A prospective study was performed to explore patients' experience of BOT, recruiting women with vulval LS from the department's monthly vulval clinic, which manages up to 250 women with vulval LS per year. These women usually have relatively severe disease, and are discharged to primary care once remission has been achieved. Inclusion criteria were (i) clinical diagnosis of vulval LS by a consultant dermatologist, (ii) under the care of a consultant dermatologist, (iii) in women over 18 years of age, (iv) who spoke English and were capable of completing the questionnaire. Ethical approval was granted by the Clinical Research Ethics Committee of the Cork Teaching Hospitals (reference ECM 4q 10/03/2020). Patients were invited to complete a Treatment Burden Questionnaire (TBQ),^4^ which was adapted for use in vulval LS. The TBQ is a questionnaire that has been validated for use in any chronic disease, assessing the burden of various treatments, associated financial burden, access to healthcare, and relationships with healthcare workers.^4^ It is composed of 15 items, each rated from 0 (not a problem) to 10 (significant problem) giving a global score ranging from 0 to 150, permitting categorisation into low (<36), moderate (36–65) and high BOT (>65).^5^

Thirty‐five patients with vulval LS were included. Mean age was 60.8 years (range 32–78 years). Mean time since diagnosis of vulval LS was 5 years. No patients had a history of vulval intraepithelial neoplasia or squamous cell cancer. All patients were using clobetasol propionate 0.05% topically; 23% daily (as part of an induction regimen), 26% on alternate days, 31% twice‐weekly, 14% weekly and 6% on an ‘as needed’ basis. Most patients had no other medical conditions, while over a quarter (28.6%) had hypothyroidism (Table 1).

**TABLE 1:** Current therapy and co‐morbidities of patients included in the study

		*n*(%)
Treatment	Daily CP	8 (22.9%)
	Alternate daily CP	9 (25.7%)
	Twice weekly CP	11 (31.4%)
	Weekly CP	5 (14.3%)
	PRN CP	2 (5.7%)
Co‐morbidities	None	18 (51.4%)
	Hypothyroidism	10 (28.6%)
	Dyslipidemia	5 (14.3%)
	Hypertension	5 (14.3%)
	Gastro‐esophaegeal reflux disease	4 (11.4%)
	Ischaemic heart disease	3 (8.6%)
	Vitamin B12 deficiency	3 (8.6%)
	Osteoporosis	2 (5.7%)
	Rheumatoid arthritis	1 (2.9%)
	Type one diabetes mellitus	1 (2.9%)
	Asthma	1 (2.9%)
	Atrial fibrillation	1 (2.9%)

*Note*: CP, clobetasol propionate 0.05%, PRN, pro re nata (as required).

Mean Dermatology Life Quality Index (DLQI) score was 4.9 (range 0–19). Mean BOT score was 33.2/150 (range 0–90). Nineteen (54.3%) achieved a score of 35 or less, signifying low BOT; ten (28.6%) between 36 and 65, signifying moderate BOT; and six (17.1%) above 65, signifying high BOT (Figure 1). Seven (20%) patients reported BOT scores of greater than 59, which has been designated as a cut‐off for increased risk of treatment‐related burnout,^5^ indicating that these patients will be unable to sustain their treatment burden over time. Surprisingly, frequency of treatment with TCS was not associated with increased BOT (*r* = 0.019, p 0.92). Of the six patients who reported high BOT scores, two were treating daily, three were treating twice weekly, and one weekly. Higher BOT scores were moderately correlated with higher DLQI scores (*r* = 0.47, *p* < 0.01). 10 of 11 patients reporting moderate‐high impact DLQI scores also recorded moderate‐high impact BOT scores. There was no correlation between duration of disease and BOT (*r* = 0.14, *p* = 0.42). Patients with hypothyroidism had a mean score of 28.9 and patients with no history of hypothyroidism had a mean score of 33.2 (*p* = 0.35).

**FIGURE 1:**
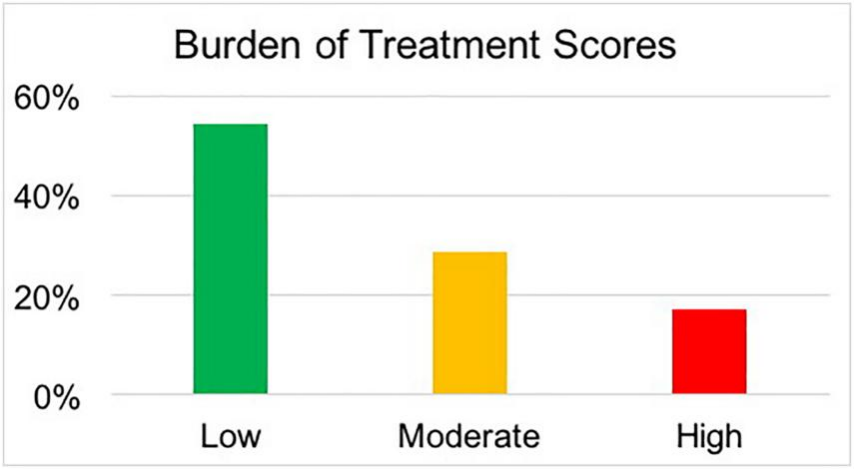
Results of Treatment Burden Questionnaire scores, divided into mild (<35, *n* = 19), moderate (36–65, *n* = 10), and high (>66, *n* = 6) categories

BOT is an important consideration in management guidelines for all chronic conditions, to help improve treatment decisions, support and avoid over‐burdening the patient, and ultimately improve treatment outcomes.^6^ This study shows that most women with vulval LS have low BOT. This is not surprising, as treatment is based around relatively infrequent topical therapy, without need for systemic therapy and associated monitoring. However, a minority are at risk of treatment‐related burnout. Future research should explore the reasons for higher BOT in these patients, as it was not related to frequency of therapy, but was related to quality of life. Our group has previously explored patient adherence to TCS in vulval LS, and concerns regarding safety of TCS are the most commonly reported reasons for non‐adherence to treatment.^7^ These concerns often stem from interactions with other healthcare professionals, such as general practitioners or pharmacists, who may provide advice that is discrepant with the treatment plan prescribed by the specialist managing their condition.^8^ We hypothesise that patients with higher BOT scores may harbour concerns about potent TCS (‘steroid phobia’), which may be inappropriately reinforced by other healthcare professionals, and this cognitive dissonance may lead to under treatment of their condition. Untreated vulval LS itself can have a significant impact on a patient's quality of life^1^, ^2^ and therefore treatment is important to minimise this. Most patients in this study had no co‐morbidities, but patients who have developed complications associated with vulval LS, such as VIN or SCC, or associated autoimmune conditions such as hypothyroidism, may have higher BOT related to management of these associated diseases. There were no patients in our study with a history of VIN/SCC, and there was no significant difference in this study between patients who had hypothyroidism, and those who did not, although the study was not powered to stratify according to thyroid status.

To our knowledge, BOT has never previously been explored in vulval LS. Our monocentric study, involving patients in a specialised dermatology clinic, was limited by its small sample size. Patients who are cared for in primary care or gynaecology may have a different spectrum of BOT. The small number of patients in the study may also preclude the generalisation of results to other patients with vulval LS. However, it illustrates that, although lower than in some other chronic conditions such as atopic dermatitis or diabetes mellitus,^9^, ^10^ BOT can be significant for some patients with vulval LS. Future research should explore the BOT in vulval LS using qualitative methods, to more deeply characterise the reasons for variance in BOT.

We recommend that dermatologists and other health care professionals who manage vulval LS should educate and reassure other healthcare professionals and all patients with vulval LS about the safety of TCS, and BOT should be considered when formulating therapeutic strategies for vulval LS.

## CONFLICT OF INTEREST

None.

## AUTHOR CONTRIBUTIONS

**Ciara O'Grady:** Data curation‐Equal, Formal analysis‐Equal, Investigation‐Lead, Project administration‐Supporting, Software‐Equal, Visualization‐Equal, Writing – original draft‐Equal, Writing – review & editing‐Supporting. **Catha, O'Connor:** Conceptualization‐Equal, Data curation‐Equal, Formal analysis‐Equal, Investigation‐Supporting, Methodology‐Lead, Project administration‐Equal, Resources‐Equal, Software‐Equal, Supervision‐Equal, Validation‐Equal, Visualization‐Equal, Writing – original draft‐Equal, Writing – review & editing‐Lead, **Aysha Al Moosa:** Data curation‐Equal, Project administration‐Equal, Writing – original draft‐Supporting, **Michelle Murphy:** Conceptualization‐Equal, Investigation‐Equal, Methodology‐Equal, Project administration‐Equal, Resources‐Equal, Supervision‐Equal, Writing – review & editing‐Equal, **Eilis Nic Dhonncha:** Conceptualization‐Equal, Data curation‐Equal, Formal analysis‐Equal, Investigation‐Equal, Methodology‐Equal, Project administration‐Equal, Resources‐Equal, Software‐Equal, Supervision‐Equal, Validation‐Equal, Visualization‐Equal, Writing – original draft‐Equal, Writing – review & editing‐Equal.

## Data Availability

Available on request.
